# MiR-122 Participates in Oxidative Stress and Apoptosis in STZ-Induced Pancreatic *β* Cells by Regulating PI3K/AKT Signaling Pathway

**DOI:** 10.1155/2021/5525112

**Published:** 2021-05-12

**Authors:** Jing Wang, Zhichun Dong, Liyin Lou, Lijuan Yang, Jingying Qiu

**Affiliations:** ^1^Department of Endocrinology, Rheumatism and Immunology, Shengzhou People's Hospital, The First Affiliated Hospital of Zhejiang University Shengzhou Branch, Zhejiang, Shengzhou 312400, China; ^2^Department of Endocrine and Metabolic Diseases, The First Affiliated Hospital of Wenzhou Medical University, Zhejiang, Shengzhou 312400, China

## Abstract

At present, there are few reports concerning the relationship between miR-122 and diabetes. In addition, the effect of miR-122 on streptozotocin- (STZ-) induced oxidative damage in INS-1 cells remains unclear. The present study aimed to investigate the role and modulatory mechanisms involving miR-122 in diabetes. STZ was used to induce INS-1 cell damage. Reverse transcription-quantitative PCR was used to investigate the expression of miR-122. A TUNEL cell apoptosis detection kit was used to detect apoptosis. Intracellular ROS levels were determined using dichlorofluorescein-diacetate. The activities of insulin secretion, superoxide dismutase (SOD), catalase (CAT), and glutathione peroxidase (GSH-px) were measured using ELISA kits. Western blotting was used to measure the expression levels of Bax, Bcl-2, PI3K, p-PI3K, caspase-3 and caspase-9, cleaved-caspase-3 and cleaved-caspase-9, AKT, and p-AKT. Then, LY294002 (LY, PI3K inhibitor) was used to treat INS-1 cells, and oxidative stress and apoptosis were measured. The results showed that STZ-induced inhibitory effects on insulin secretion were mitigated by miR-122 inhibitor, and the activities of SOD, CAT, and GSH-px were also increased. Furthermore, miR-122 inhibitor inhibited apoptosis and oxidative stress in STZ-induced INS-1 cells. Finally, the addition of LY increased insulin levels; reduced the activities of SOD, CAT, and GSH-px; and promoted apoptosis in STZ-induced INS-1 cells. In conclusion, interference with miR-122 can inhibit oxidative stress and apoptosis in STZ-induced INS-1 cells, involving a mechanism of action related to the PI3K/AKT pathway.

## 1. Introduction

MicroRNAs (miRNAs) are a type of noncoding single-stranded RNA comprising small molecules 18–25 nucleotides in length [1] that mainly promote the degradation of mRNA or inhibit target messenger RNAs (mRNAs) through complementary base pairing with downstream target gene mRNA [2]. miRNAs are of diverse types and have complex functions that are widely involved in regulating the proliferation and differentiation of various cells [3], and dysregulation of expression can promote or inhibit tumor development. miR-122 is a type of miRNA with tumor suppressor properties [4]. It has been confirmed that miR-122 exhibits low expression in liver cancer [5] and breast malignancies [6], whereas there are a few studies concerning relationships involving miR-122 and pancreatic *β* cells.

Diabetes mellitus (DM) is a chronic metabolic disease that currently affects > 400 million people worldwide [7]. By 2030, the number of diabetics is expected to surge to 439 million [8]. Type 2 diabetes mellitus (T2DM) accounts for 90% of all diabetes cases. The International Diabetes Federation announced that 336 million people worldwide currently suffer from T2DM and that the disease is responsible for 4.6 million deaths each year, or one death every seven seconds [9]. Pancreatic *β*-cells are responsible for insulin secretion and control of plasma glucose levels [10]. There is increasing evidence of a correlation between cell dysfunction/death and the onset of diabetes [11–13]. In the prediabetes stage of T2DM, pancreatic *β*-cell function is already diminished [14]. As the course of the disease progresses, the long-term high-glucose environment further damages the *β*-cells, leading to a gradual decline in insulin secretion and cellular apoptosis [15]. Improving the function of pancreatic *β*-cells and inhibiting *β*-cell apoptosis may be important targets to inhibit the occurrence and development of T2DM.

Thus, the purpose of this study was to investigate potential interactions involving miR-122 and pancreatic *β*-cells and to determine their effects on pancreatic *β*-cell activities, such as cell proliferation and apoptosis.

## 2. Materials and Methods

### 2.1. Cell Culture and Cell Model Construction

INS-1 (Cell Resource Center, Institute of Basic Medicine, Chinese Academy of Medical Sciences, Peking, China) cells were maintained in RPMI 1640 (Gibco, CA, USA) containing 11 mM glucose, 10% (v/v) fetal bovine serum (FBS) (10099141, Gibco, CA, USA), 50 *μ*M *β*-mercaptoethanol (M3148, Sigma, St. Louis, Missouri, USA), and 10 mM HEPES (H1090, Solarbio, Beijing, China) at 37°C under 5% CO_2_. INS-1 cells were treated with STZ (streptozotocin, 3 mmol/L, Sigma, St. Louis, Missouri, USA) for 12 and 24 h.

### 2.2. Transfection of miR-122 Inhibitor

MicroRNA-negative control (miRNA-NC) and miR-122 inhibitor were purchased from Biomics Biotechnologies (Nantong, China). Cell transfection was performed using Lipofectamine 2000 (Invitrogen, CA, USA) according to the manufacturer's instructions.

### 2.3. Reverse Transcription-Quantitative PCR (RT-qPCR)

Total RNA was extracted from the cell samples. First, it was extracted via Trizol® reagent (Thermo Fisher Scientific, Inc., CA, USA), then reverse-transcribed into cDNA using a commercial RevertAid™ cDNA Synthesis kit from Takara Bio, Inc. (Dalian, China) at 42°C for 1 h, and inactivated at 90°C for 5 min. SYBR Premix Ex Taq™ II kit (Thermo Fisher Scientific, Inc.) was used for qPCR. The PCR mixture contained 3 mM MgCl_2_, 0.5 *μ*M forward and reverse primers, 2 *μ*L SYBR Green PCR master mix, and 2 *μ*L cDNA. Samples were amplified on a QuantStudio 3 Real-Time PCR system (Applied Biosystems; Thermo Fisher Scientific, Inc.). Thermocycling conditions for qPCR were 5 min at 95°C, with 40 cycles of 30 s at 95°C and 45 s at 65°C. Expression levels were normalized to those of U6. The primers used were as follows:

miR-122 forward 5′-GCAGGTCAGTGATCTG-GATTCG-3, miR-122 reverse 5-GCAGGCTTCGATGCATTGT-3′; U6 forward, 5′-CTCGCTTCGGCAGCACATATA-3 U6 reverse 5′-ACGCTTCACGAATTTGAGTGTC-3′.

### 2.4. Glucose-Stimulated Insulin Secretion

INS-1 cells were seeded at a density of 1 × 10^5^ cells/well in a 24-well plate, with a control group (normal INS-1 cells), STZ cell group (cells treated with STZ), STZ + inhibitor-NC (cells treated with STZ and inhibitor-NC), and STZ + miR-122 inhibitor (cells treated with STZ and miR-122 inhibitor). After 24 h, cells were washed and incubated with Krebs–Ringer bicarbonate HEPES buffer (KRBH, 135 mM NaCl, 3.6 mM KCL, 5 mM NaHCO_3_, 0.5 mM NaH_2_PO_4_, 0.5 mM MgCl_2_, 1.5 mM CaCl_2_, and 10 mM HEPES, pH 7.4, and 0.1% bovine serum albumin) (Sigma, St. Louis, Missouri, USA), 3.3 mM glucose (hypoglycemic glucose concentration), and supernatants at different dilutions (37°C, 30 min) and subsequently stimulated for 1 h with 16.7 mM glucose. At the end of each INS-1 stimulation, the medium was collected, cleared by centrifugation, and stored at −80°C for subsequent analysis. ELISA kits (Beyotime Institute of Biotechnology, Beijing, China) were used to assay the insulin content of supernatants.

### 2.5. Dichlorofluorescein Staining to Determine Cell ROS Levels

INS-1 cells were seeded at a density of 2 × 10^5^ cells/well in a 6-well plate. The control group is divided into Group Control (normal INS-1 cells), Group STZ (cells treated with STZ), Group STZ+inhibitor-NC (cells treated with STZ and inhibitor-NC), and Group STZ+miR-122 inhibitor (cells treated with STZ and miR-122 inhibitor). After 24 h, the kit (Reactive Oxygen Species Assay Kit, Shanghai Yisheng Bio-Technology Co., Ltd. China) was used according to the instructions. The culture medium was discarded and cells were washed once with PBS (Beyotime Institute of Biotechnology). Dichlorofluorescein-diacetate was diluted in a serum-free culture medium (Gibco; Thermo Fisher Scientific) at a ratio of 1 : 1000 to a final concentration of 10 *μ*M. Cells were added to a 6-well plate (1000 *μ*L/well) and incubated with cells for 30 min (37°C, 5% CO_2_). The culture medium was discarded and cells were washed with serum-free culture medium 3 times. Observed images (×400) were acquired under a fluorescence microscope (Olympus Corporation, Tokyo, Japan).

### 2.6. Determination of Oxidative Stress

INS-1 cells were collected and homogenized (10% *w*/*v*) in cold saline. The activity levels of superoxide dismutase (SOD; cat. no. A001-1-2), catalase (CAT; cat. no. A007-1-1), and glutathione peroxidase (GSH-Px; cat. no. A005-1-2) were determined using colorimetric commercial kits (Nanjing Jiancheng Bioengineering Institute, Nanjing, China) according to the manufacturer's protocols.

### 2.7. Terminal Deoxynucleotidyl Transferase-Mediated dUTP-Biotin Nick End Labeling Assay

INS-1 cells were seeded at a density of 2 × 10^5^ cells/well in a 6-well plate. The cells were divided into the following groups: control group (normal INS‐1 cells), STZ (cells treated with STZ), STZ + inhibitor-NC (cells treated with STZ and inhibitor-NC), STZ + miR-122 inhibitor (cells treated with STZ and miR-122 inhibitor), and STZ + LY (a PI3K inhibitor, Beyotime Institute of Biotechnology) + miR-122 inhibitor (cells treated with STZ, LY294002, and miR-122 inhibitor). After 24 h, the culture medium was discarded, and cells were washed once with PBS (Beyotime Institute of Biotechnology). Cells were fixed with immunostaining fixative (Beyotime Institute of Biotechnology) at 25°C for 30 min and washed with PBS once. After adding the immunostaining permeabilization solution, incubation was conducted for 5 min at room temperature, and the cells were washed twice with PBS. TUNEL detection solution was added to cells and incubated for 60 min at 37°C in the dark. Cells were washed with PBS 3 times and nuclei counterstained with DAPI (Beyotime). The stained slides were observed under a light microscope (Olympus Corporation, ×100).

### 2.8. Western Blotting

Cells were harvested and total protein was extracted using RIPA lysis buffer (Beyotime Institute of Biotechnology, China). Protease inhibitor (Beyotime) was added to the lysis buffer (1 : 100). The lysates were centrifuged at 4°C at 850 ×g for 15 min. The supernatant was collected and mixed with loading buffer (Beyotime) containing 100 mM dithiothreitol. Total protein was quantified using a Protein Concentration Determination Kit (Beyotime), and proteins (30 *μ*g/lane) were separated by 15% SDS-PAGE. The separated proteins were subsequently transferred onto PVDF membranes (EMD Millipore, Darmstadt, Germany) and blocked with 5% BSA (Beyotime) at room temperature for 2 h. The membranes were then incubated with the following primary antibodies (Abcam, Cambridge, UK) at 4°C overnight: Anti-Bcl-2 (cat. no. ab32124; 1 : 1,000 dilution), anti-Bax (cat. no. ab32503; 1 : 1,000 dilution), anti-caspase-3 (cat. no. ab2302; 1 : 1,000 dilution), anti-caspase-9 (cat. no. ab32539; 1 : 1,000 dilution), anti-AKT (cat. no. ab38449; 1 : 1,000 dilution), anti-pan-AKT (cat. no. ab8805; 1 : 1,000 dilution), anti-PI3K (cat. no. ab32089; 1 : 1,000 dilution), anti-phospho-PI3K (cat. no. ab138364; 1 : 1,000 dilution), and anti-GAPDH (cat. no. ab8245; 1 : 2,000 dilution).

### 2.9. Statistical Analysis

Data are presented as mean ± standard deviation (SD) and were analyzed using SPSS v.19.0 and GraphPad Prism 6. Comparisons between groups were performed using Student's *t*-test. For multiple comparisons, one-way ANOVA followed by Tukey's post hoc test was carried out to analyze differences. *P* < 0.05 indicated statistically significant differences.

## 3. Results

### 3.1. miR-122 Participates in Oxidative Stress in STZ-Induced Pancreatic *β* Cells

**Figure 1:**
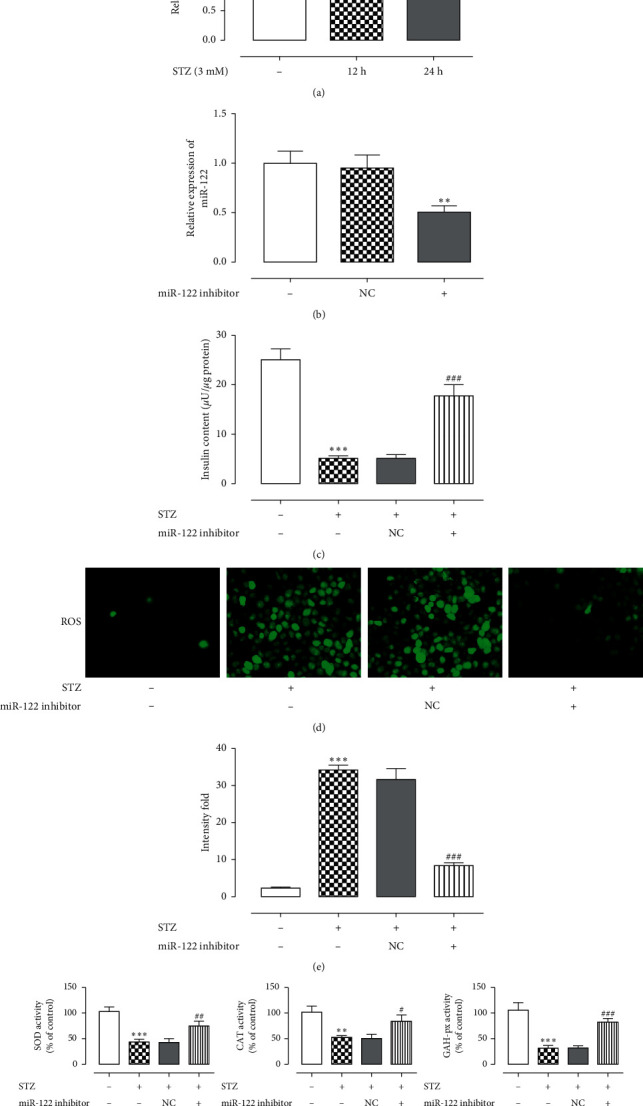
MiR-122 participates in the oxidative stress in STZ-induced pancreatic *β* cells. (a) The expression level of miR-122 in INS-1 cells detected by RT-qPCR. (b) Detection of the expression level of miR-122 after adding miR-122 inhibitor. (c) ELISA kit detects Insulin content. (d, e) Detection of the intracellular ROS level. (f) Detection of oxidative stress level in cells. ^*∗*^*P* < 0.05, ^*∗∗*^*P* < 0.01, ^*∗∗∗*^*P* < 0.001 versus control group; ^#^*P* < 0.05, ^##^*P* < 0.01, ^###^*P* < 0.001 versus STZ group. The experiment was repeated three times.

To study whether miR-122 plays a role in STZ-induced oxidative stress in pancreatic *β* cells, the expression levels of miR-122 at 12 and 24 h were detected by RT-qPCR. The results are shown in Figure 1(a). Compared with the control group, the expression of miR-122 increased with time. Compared with the control group, insulin levels in the model group were downregulated (1-C), and ROS levels were upregulated (1-D, E). SOD, CAT, and CAH-px activities were downregulated (1-F), indicating that STZ-induced oxidative stress in pancreatic *β* cells. After adding miR-122 inhibitor (1-B), compared with the control group, the insulin content increased; ROS levels decreased; and SOD, CAT, and CAH-px activities increased, indicating that inhibition of miR-122 can reduce STZ-induced INS-1 cell oxidative stress.

### 3.2. Inhibition of miR-122 Decreases Cell Apoptosis

**Figure 2:**
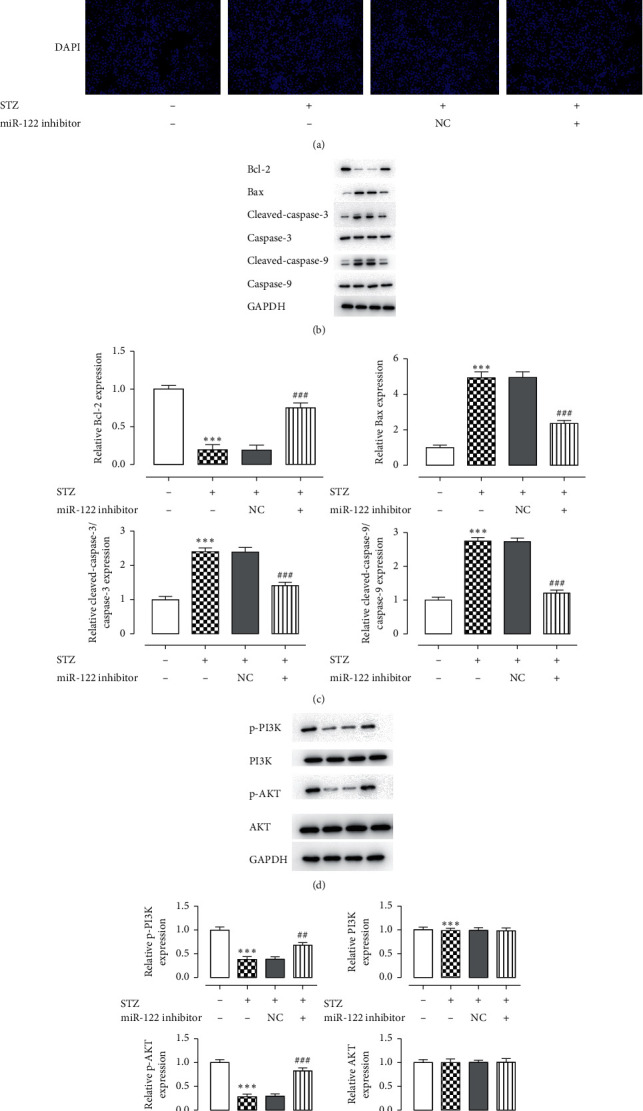
Inhibition of MiR-122 decreases cell apoptosis. (a) TUNEL staining was used to detect cell apoptosis. (b, c) Expression levels of the apoptosis-related proteins Bcl-2, Bax, cleaved caspase-3, cleaved caspase-9, procaspase-3, and procaspase-9 were analyzed using western blotting. (d, e) Interference with miR-122 activates the pI3K/AKT signaling pathway in pancreatic *β* cells induced by STZ. ^*∗*^*P* < 0.05, ^*∗∗*^*P* < 0.01, ^*∗∗∗*^*P* < 0.001 versus control group; ^#^*P* < 0.05, ^##^*P* < 0.01, ^###^*P* < 0.001 versus STZ group. The experiment was repeated three times.

In the control group, there was less green fluorescence (Figure 2(a)), high Bcl-2 expression, low Bax, and C-caspase-3, and C-caspase-9 expression, indicating less apoptosis in the control group, while the model group exhibited higher green fluorescence and low Bcl-2 expression. The expression of Bax, and C-caspase-3, and C-caspase-9 was upregulated, indicating STZ-induced apoptosis in INS-1 cells (Figure 2(b)). After adding the miR-122 inhibitor, the green fluorescence was decreased, the expression of Bcl-2 was upregulated, and the expression of Bax, C-caspase-3, and C-caspase-9 was downregulated. This showed that miR-122 inhibitor can reduce cell apoptosis.

### 3.3. Interference with miR-122 Activates PI3K/AKT Signaling Pathway in Pancreatic *β* Cells Induced by STZ

**Figure 3:**
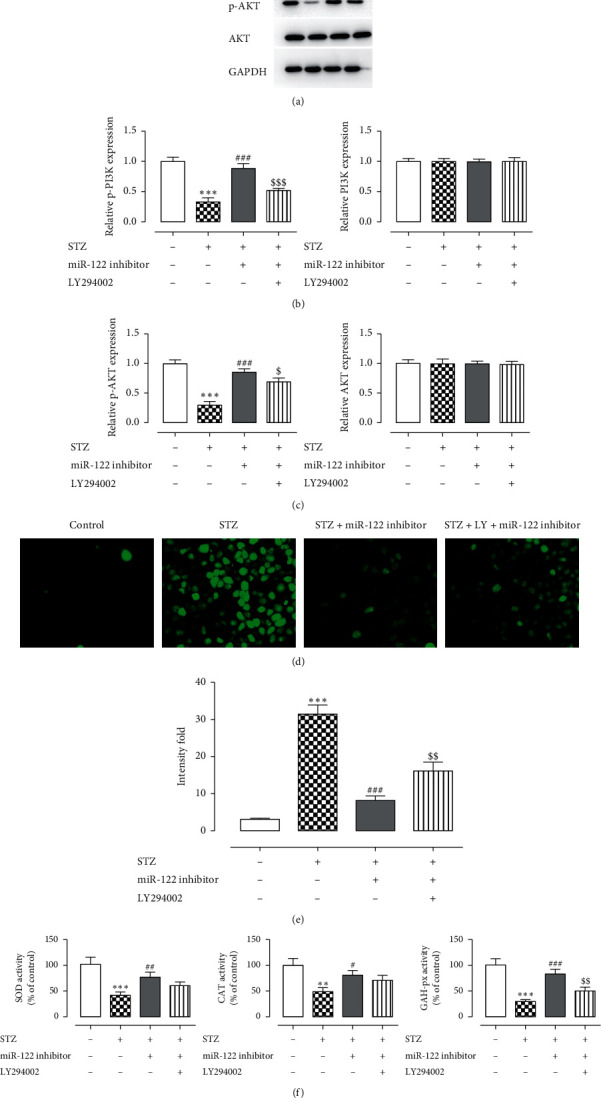
Detection of the oxidative stress levels after adding LY294002. (a-c) Detection of the expression levels of PI3K/AKT signaling pathway-related proteins after adding LY294002. (d, e) Detection of the intracellular ROS level. (f) Detection of the oxidative stress level in cells. ^*∗*^*P* < 0.05, ^*∗∗*^*P* < 0.01, ^*∗∗∗*^*P* < 0.001 versus control group; ^#^*P* < 0.05, ^##^*P* < 0.01, ^###^*P* < 0.001 versus STZ group. The experiment was repeated three times.

Western blotting was used to detect the expression levels of p-PI3K, PI3K, p-AKT, and AKT (Figure 3(a)). The expression of p-PI3K and p-AKT in the control group was upregulated, and the expression of p-PI3K and p-AKT in the model group was downregulated. Compared with the model group, the expression of p-PI3K and p-AKT was upregulated after adding miR-122 inhibitor, indicating that miR-122 is related to the PI3K/AKT signaling pathway.

### 3.4. The Expression Level of PI3K/AKT Protein Was Downregulated after LY294002 Treatment

Western blotting was used to detect the expression levels of PI3K/AKT signaling pathway-related proteins after adding LY (Figure 3). Compared with the control group, the expression of p-PI3K and p-AKT was higher in the miR-122 inhibitor group. Compared with the miR-122 inhibitor group, the expression was downregulated after the addition of LY294002 (LY), indicating that interference with the PI3K/AKT signaling pathway was successful.

### 3.5. Cell Oxidative Stress Levels Increase after Adding LY294002

The control group showed less green fluorescence, and the model group showed more green fluorescence. Green fluorescence increased after adding STZ, but instead decreased upon miR-122 inhibitor treatment, and again enhanced in the presence of LY294002 cotreatment. ROS levels are shown in Figure 3(d), which indicated that inhibiting the PI3K/AKT signaling pathway increased ROS abundance. In Figure 3(f), compared with the miR-122 inhibitor group, the activities of SOD, CAT, and GAH-px decreased after adding LY, indicating that inhibiting the PI3K/AKT signaling pathway could enhance cellular oxidative stress.

### 3.6. Apoptosis Increased after Adding LY294002

**Figure 4:**
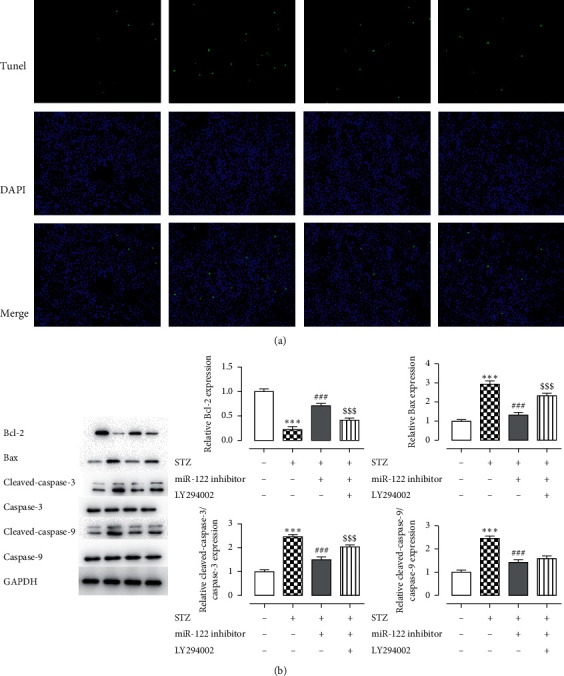
Detection of the apoptosis in INS-1 cells after adding LY294002. (a) TUNEL staining was used to detect cell apoptosis. (b) Expression levels of the apoptosis-related proteins Bcl-2, Bax, cleaved caspase-3, cleaved caspase-9, procaspase-3, and procaspase-9 were analyzed using western blotting. ^*∗*^*P* < 0.05, ^*∗∗*^*P* < 0.01, ^*∗∗∗*^*P* < 0.001 versus control group; ^#^*P* < 0.05, ^##^*P* < 0.01, ^###^*P* < 0.001 versus STZ group. The experiment was repeated three times.

The control group exhibited less green fluorescence, and the model group showed more green fluorescence (Figure 4(a)). STZ treatment obviously increased Tunel-positive cells, which decreased by miR-122 inhibitor and enhanced by LY294002, indicating that inhibiting the PI3K/AKT signaling pathway could increase cell apoptosis. Compared with the miR-122 inhibitor group, after adding LY, the expression of Bcl-2 was downregulated, and the expression of Bax, C-caspase-3, and C-caspase-9 was upregulated (Figure 4(b)), indicating that the inhibition of the PI3K/AKT signaling pathway increased INS-1 cell apoptosis.

## 4. Discussion

Diabetes is an endocrine and metabolic disease characterized by high blood glucose levels [16]. At present, it is believed that the pathogenesis of diabetes is mainly caused by the apoptosis and dysfunction of *β* cells, which leads to decreased insulin secretion [17]. In the experiments reported here, STZ was used to induce pancreatic *β*-cell (INS-1) damage. Oxidative stress was measured via the activity levels of insulin, ROS, SOD, CAT, and GSH. Apoptosis of INS-1 cells was detected by TUNEL staining and Bax and Bcl-2 proteins.

Oxidative stress refers to the imbalance between oxidation and antioxidation in the body after stimulation. A large number of oxidative intermediates are produced, which leads to body damage [18]. ROS is a metabolite of redox reactions in the body, which has the effect of scavenging free radicals [19]. When the body is in pathological states, such as diabetes, the functions of mitochondria are diminished, and the structural integrity of the cell membrane is abolished, with a large amount of ROS produced [20].

As shown in Figure 1, the control group showed less green fluorescence, indicating a low ROS content, while the STZ-induced model group showed more green fluorescence, indicating higher ROS levels. ROS content in the miR-122 inhibitor group decreased, indicating that miR-122 could inhibit ROS levels. SOD, GSH-P x, and CAT are antioxidant enzymes. When their content in the body is reduced, the free radical clearance rate is reduced, which leads to oxidative stress [21]. As shown in Figure 1, the levels of SOD, CAT, and GSH-Px were higher in the control group, while the contents in the model group were significantly reduced. Increased activity was identified in the STZ + miR-122 inhibitor group, indicating that miR-122 could enhance the activity of SOD, CAT, and GSH-Px. Therefore, our experiments have shown that miR-122 inhibitor can inhibit cellular oxidative stress.

Apoptosis is a specific mode of cell death with a characteristic pattern of morphological, biochemical, and molecular changes [22]. Apoptosis is closely regulated by the B-cell lymphoma 2 (Bcl-2) family and by the caspase family of intracellular proteins [23]. The caspase family is a central component of the machinery responsible for apoptosis [24]. The Bcl-2 family regulates both pro- and antiapoptotic proteins by mediating the permeabilization of the mitochondrial membrane, and Bcl-2 proteins serve as an “apoptotic switch” [25]. Our TUNEL experiments showed that miR-122 had an inhibitory effect on apoptosis (Figure 2). Western blotting experiments revealed that, in the STZ + miR-122 inhibitor group, the expression of Bax, C-caspase-3, and C-caspase-9 was downregulated, and the expression of Bcl-2 was increased, indicating that miR-122 could inhibit cell apoptosis.

Oxidative stress and apoptosis of INS-1 cells were detected after the addition of LY294002 (LY). As shown in Figure 4, the ROS content of the STZ + miR-122 inhibitor group was increased, while the activities of SOD, CAT, and GSH-Px decreased, indicating that miR-122 regulates oxidative stress in INS-1 cells through the PI3K/AKT signaling pathway. Figures 3 and 4 show that after the addition of LY, compared with the STZ + miR-122 inhibitor group, apoptosis was increased, and increased apoptotic protein contents indicated that miR-122 regulated oxidative stress in INS-1 cells through the PI3K/AKT signaling pathway.

## 5. Conclusions

In summary, miR-122 inhibits oxidative stress and apoptosis of INS-1 cells, and its regulation is related to the PI3K/AKT signaling pathway. However, there are limitations to the present study. First, the study was an *in vitro* one, and no *in vivo* experiments were performed. Second, the molecular mechanisms underlying the effects of miR-122 inhibition by the PI3K/AKT signaling pathway on INS-1 cell function were not fully investigated. These issues require further in-depth investigations and will be addressed in future studies.

## Data Availability

The datasets used and/or analyzed during the current study are available from the corresponding author on reasonable request.
